# Beneficial Effects of Fermented Papaya Preparation (FPP^®^) Supplementation on Redox Balance and Aging in a Mouse Model

**DOI:** 10.3390/antiox9020144

**Published:** 2020-02-07

**Authors:** Mariantonia Logozzi, Rossella Di Raimo, Davide Mizzoni, Mauro Andreotti, Massimo Spada, Daniele Macchia, Stefano Fais

**Affiliations:** 1 Department of Oncology and Molecular Medicine, Istituto Superiore di Sanità, Viale Regina Elena 299, 00161 Rome, Italy; mariantonia.logozzi@iss.it (M.L.); rossella.diraimo@iss.it (R.D.R.); davide.mizzoni@iss.it (D.M.); 2 National Center for Global Health, Istituto Superiore di Sanità, Viale Regina Elena 299, 00161 Rome, Italy; mauro.andreotti@iss.it; 3 Centro Nazionale Sperimentazione e Benessere Animale, Istituto Superiore di Sanità, Viale Regina Elena 299, 00161 Rome, Italy; massimo.spada@iss.it (M.S.); daniele.macchia@iss.it (D.M.)

**Keywords:** FPP^®^, nutraceutical supplementation, C57BL/6J, anti-aging effect, antioxidant effect, telomeres, telomerase, SOD-1, GSH

## Abstract

In recent decades much attention has been paid to how dietary antioxidants may positively affect the human health, including the beneficial effects of fermented foods and beverages. Fermented Papaya Preparation (FPP^®^) has been shown to represent a valuable approach to obtain systemic antioxidants effect. In this study, we wanted to verify whether FPP^®^ had a clear and scientifically supported in vivo anti-aging effect together with the induction of a systemic antioxidant reaction. To this purpose we daily treated a mouse model suitable for aging studies (C57BL/6J) with FPP^®^-supplemented water from either the 6th weeks (early treatment) or the 51th weeks (late treatment) of age as compared to mice receiving only tap water. After 10 months of FPP^®^ treatment, we evaluated the telomerase activity, antioxidants and Reactive Oxygen Species ROS plasmatic levels and the telomeres length in the bone marrow and ovaries in both mice groups. The results showed that the daily FPP^®^ assumption induced increase in telomeres length in bone marrow and ovary, together with an increase in the plasmatic levels of telomerase activity, and antioxidant levels, with a decrease of ROS. Early treatment resulted to be more effective, suggesting a potential key role of FPP^®^ in preventing the age-related molecular damages.

## 1. Introduction

Fermentation is one the most ancient methods of food preparation that exploits the growth and metabolic activities of microorganisms to preserve and transform food materials [1,2]. During this process, growth of spoilage and pathogenic organisms is inhibited by secondary metabolites produced by fermenting organisms, preserving and extending the storage of perishable foods [3,4]. Beyond conservation, fermentation gives to food a characteristic aroma and taste and enhance their organoleptic profiles and palatability, digestibility of proteins and carbohydrates, and the bioavailability of vitamins and minerals [4,5,6,7]. Since ancient times, fermented foods and beverages have been a fundamental part of human diet and their beneficial effects in reducing cholesterol levels and blood pressure, boosting immune system, protecting from toxic pathogens and in the prevention of carcinogenesis, osteoporosis, diabetes, cardiovascular and hepatic diseases have been widely characterized [8]. Moreover, fermented foods and beverages have a strong impact on human gut microbiota. It is well established that intestinal bacteria modulate the metabolic profile of the host, also influencing the immune system [9,10] and maintaining the structure and function of the intestinal tract [11,12]. The interactions between ingested fermented food and microbiota constitute a rapidly expanding field of study, focusing in particular on human health impact [12,13,14,15,16]. 

More than 100 years ago Metchnikoff, Nobel prize winner for the discovery of macrophages, already claimed that the longevity of some populations of Eastern Europe was due to the high quantity of fermented food in their diet [17] and more recently it has been shown that microbiota in the elderly is strongly influenced by diet, opening up that healthy ageing is associated with microbial diversity [18,19]. 

Fermented Papaya Preparation (FPP^®^, Immun’Âge^®^) is one of the fermented foods that have proven positive effects on brain health; FPP^®^ is a product resulting from yeast fermentation of non-genetically modified *Carica Papaya* Linn, which is marketed as a natural dietary functional health supplement under the brand name of Immun’Âge^®^ [20,21]. FPP^®^ is a powerful antioxidant and nutraceutical adjuvant in combined therapies against various diseases [22,23,24,25,26,27,28,29], including cancer [22,25,30]. The FPP^®^ more documented actions are as a free radical regulator [31], as immunomodulator [32,33,34,35,36] and as antioxidant [37,38]. In fact, FPP^®^ has shown a powerful *in vitro* anti-oxidative activity on brain cells [39], as well on *in vivo* experimental model of epilepsy consistently reducing neural release of epileptogenic monoamine [40]. Moreover, FPP^®^ showed a clear action in reducing the derangement of oxidant/antioxidant balance at the brain level in elderly rats and in experimental ischemia-reperfusion model [20,41,42]; FPP^®^ modulates oxidative DNA damage, protecting brain from oxidative damage in hypertensive rats and reducing genotoxic effect of H_2_O_2_ [43], and protecting the body from the aging-related diseases [44,45,46,47], including neurodegenerative diseases [47,48,49]. However, a clear *in vivo* action of FPP^®^ on the molecular signature of aging, such as telomerase activity and telomeres length has not been provided yet.

In this study we investigated the role of in vivo FPP^®^ administration in the induction of an antioxidant action together with an anti-aging effect. The experimental design provided that the mice received the FPP^®^ in water either from 6 weeks (ET-FPP^®^ group: early treatment with FPP^®^), or from 51 weeks of life (LT-FPP^®^ group: late treatment with FPP^®^), as compared to mice receiving FPP^®^-free tap water (CTR group). For both treatment groups, at the end of treatment period (10 months), we evaluated antioxidants (Total Antioxidant Capacity, SOD-1 and GSH), ROS and telomerase activity levels in blood samples, and telomeres length in single cell suspensions from the bone morrow and the ovaries of the mice. Our results showed the effect of FPP^®^ in inducing a clear systemic antioxidant reaction (higher of SOD-1 and GSH plasma levels) along with an increased telomerase activity and longer telomeres in both the bone marrow and the ovaries of the treated mice. Lastly, FPP^®^ was more effective when it starts at an early age as compared to late treatment.

## 2. Materials and Methods

### 2.1. Immun’Âge^®^-FPP^®^ (Fermented Papaya Preparation)

The FPP^®^ (Immun’Âge^®^, patent number 6401792, Osato Research Institute, Gifu, Japan), used in the present study was obtained from *Carica papaya* L. cultivated in Hawaii, followed by yeast fermentation for 10 months and batch-to-batch checking at the Osato Research Institute. FPP^®^ was dissolved in tap water and administrated every day without interruption. 

### 2.2. In Vivo Studies

**Figure 1:**
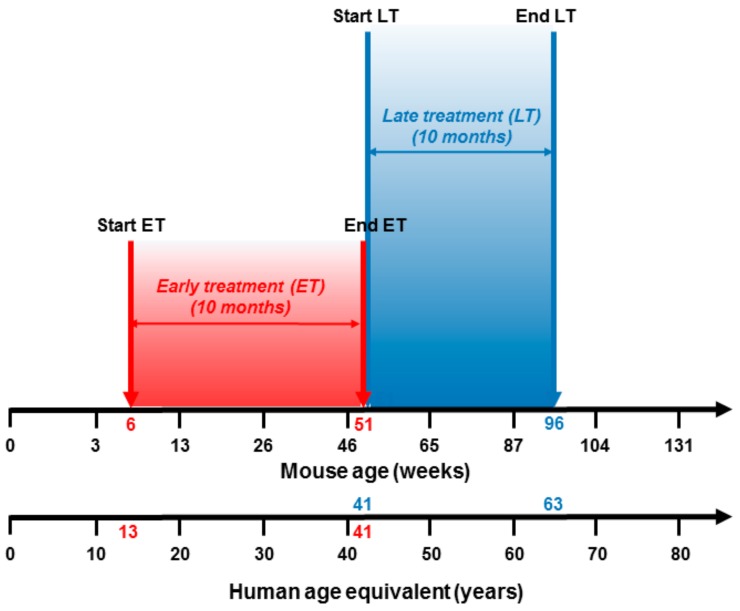
Equivalence between mice age and human age. Early treatment of mice from 6 weeks to 51 weeks of age corresponds to treatment in humans from 13- to 41-years old. Late treatment of mice from 51 weeks to 96 weeks of age corresponds to treatment in humans from 41- to 63-years old.

For our analysis, we have chosen an aging female mouse model (C57BL/6J), in order to have available cells from organs with either gender-independent (i.e., the bone marrow) or gender-dependent (i.e., ovaries) functions, and divided mice into two groups: FPP^®^ was daily administered to the first group for 10 months from 6 weeks old (6 to 51 weeks of age) (ET-FPP^®^: early treatment with FPP^®^) and to the second group for 10 months from 51 weeks old (51 to 96 weeks of age) (LT-FPP: late treatment with FPP^®^); in both conditions a control group was included receiving tap water only (ET-CTR and LT-CTR). Each group consisted of 10 animals for statistical significance. To compare the mice treatment groups to the human age, ET treatment corresponded to women starting FPP® at 13 years and ending FPP^®^ at 41 years of age; while LT treatment starting at 41 years and ending at 63 years of age. (Figure 1).

Each treated mice drank 1 mL of FPP^®^-supplemented water every day, corresponding to 6 mg/mouse/day of FPP^®^. Just before mice sacrifice, blood was withdrawn from mice eyes. Immediately after the sacrifice, bone marrow was isolated from both tibias and femurs of the mice hind legs, while ovaries were retrieval from reproductive system. Blood, bone marrow cells and ovarian germ cells were used for subsequent experimental analysis of aging parameters.

All the studies were approved by the ethical committee of the Italian National Institute of Health (Rome, Italy) and were conducted in accordance with the current Italian Law (Law 26/2014), authorization n◦792/2017-PR (prot. D9997.49 27/06/2017), that regulates experiments in laboratory animals. 40 C57BL/6J female mice between 16 and 20 g (4 weeks of age) were purchased from Charles River Laboratories Italia s.r.l., (Calco, Lecco, Italy), and housed in the animal facility of the Italian National Institute of Health. Mice had 10 and 14 h periods of light and darkness respectively, were housed in a different number of animal cages, depending on the experiment, with ad libitum mice chow (Mucedola, Settimo Milanese (MI), Italy) and water intake. Accordingly to the guidelines for a correct laboratory practice and signs of poor quality of life, a veterinarian responsible for animal welfare checked mice twice a week, to monitor signs of sufferance such as weight loss, decreased water and food consumption, poor hair coat, decreased activity levels and tumor ulcerations 

### 2.3. Total Antioxidant Power Assay (PAO Test Kit)

Detection and quantification of Total Antioxidant Power was performed in FPP^®^-supplemented water using a colorimetric assay: PAO Test kit for Total Antioxidant Capacity (JaICA, Japan). The assay can detect not only hydrophilic antioxidants such as Vitamin C, glutathione, but also can detect hydrophobic antioxidants such as Vitamin E. The determination of the antioxidant power is carried out using the reduction of the cupric ion (Cu^++^ to Cu^+^). Briefly, samples were incubated for 3 min at room temperature with Cu^++^ solution, Cu^++^ are reduced by antioxidants to form Cu^+^ that reacts with chromatic solution (bathocuproine), and can be detected by absorbance at wavelength 480 to 490 nm. Antioxidant capacity can be calculated from the Cu^+^ formed. Absorbance was recorded at 490 nm.

### 2.4. Ascorbic Acid Assay

Detection and quantification of Ascorbic Acid in FPP^®^-supplemented water was performed using a fluorometric Ascorbic Acid Assay Kit (Abcam, Cambridge, UK). Samples were diluted in ascorbic acid buffer in 96-well plate and subsequently to each well was added catalyst and then reaction mix. After 3 min of incubation, fluorescence was read in microplate reader at Ex/Em = 535/590 nm.

### 2.5. Collection and Processing of Murine Plasma from Blood Samples

Blood samples collection from each group mice was performed by retro-orbital bleeding (ROB) immediately before the sacrifice. This safe phlebotomy technique allowed to obtain high-quality samples of adequate volume (500 μL/mouse) for analysis [50]. Blood samples were collected in K3-EDTA-coated collection tubes. To obtain plasma samples, EDTA-treated whole blood from each mouse was centrifuged at 400 g for 20 min. Plasma samples (250 μL/mouse) were then collected and immediately analyzed or stored at −80 °C until analysis.

#### 2.5.1. Total Antioxidant Power Assay (PAO Test kit)

Detection and quantification of Total Antioxidant Power was in mice plasma obtained before the sacrifice using a colorimetric assay: PAO Test kit for Total Antioxidant Capacity (JaICA). After centrifugation of blood at 400× *g* for 20 min, supernatant was collected and immediately analyzed. Briefly, samples were incubated for 3 minutes at room temperature with Cu^++^ solution, subsequently Stop Solution was added to each well. Absorbance was recorded at 490 nm.

#### 2.5.2. Superoxide Dismutase (SOD) Activity Assay

The Superoxide Dismutase Activity kit (Thermo Fisher, Waltham, MA, USA), a colorimetric assay, was used for detection and quantification of superoxide dismutase activity in mice plasma preparations. Plasma samples were incubated for 20 min at room temperature after the addition of the sample and substrate and chromogenic detection reagent. The optical densities were recorded at 450 nm.

#### 2.5.3. Reduced Glutathione (GSH) Detection and Quantification Assay

Glutathione Colorimetric Detection Kit (Thermo Fisher), a colorimetric assay, was used for detection and quantification of reduced glutathione (GSH) levels in plasma preparations. Detection reagent and reaction mixture were added to samples and after 20 min of incubation at room temperature the optical densities were recorded at 405 nm.

#### 2.5.4. Total Reactive Oxygen Species (ROS) Assay

Total Reactive Oxygen Species (ROS) Assay Kit 520 nm (Thermo Fisher) was used to analyze the total ROS levels in mice plasma preparations. 10 µl of each plasma sample were added to 100 µL of 1× ROS Assay Stain. After for 60 min of incubation at 37 °C and 5% CO_2_, signals were analyzed using a fluorescent microplate reader off the 488 nm (blue laser) in the FITC channel.

#### 2.5.5. Detection of telomerase by ELISA Assay

Quantitative determination of mouse telomerase concentrations was performed in plasma preparations using a colorimetric sandwich-ELISA assay, Mouse TE(telomerase) ELISA Kit (Elabsciences^®^, Houston, TX, USA. The optical density (OD) was measured at 450 ± 2 nm.

### 2.6. Bone Marrow Cells Recovery from Mice

Immediately after the sacrifice of CTR, ET-FPP^®^ and LT-FPP^®^ mice, bone marrow was obtained from both tibias and femurs of the hind legs of mice [51,52,53,54]. Bone marrow was then placed in physiological solution (NaCl) and disrupted with the blunt end of a 5-mL syringe plunger. Bone marrow cells were isolated using a Falcon^®^ 100 μm cell strainer (Corning, Corning, NY, USA), obtaining a uniform single-cell suspension from bone marrow. The single-cell suspensions were washed twice in PBS and immediately processed for following analysis.

### 2.7. Ovarian Germ Cells Recovery from Mice

Immediately after the sacrifice of CTR, ET-FPP^®^ and LT-FPP^®^ mice, ovaries were dissected [51,52,53,55], placed in physiological solution (NaCl) with 1% of trypsin and 0.1 μM of EDTA, separated from the remaining reproductive system with a cutter and disrupted with the blunt end of a 5-mL syringe plunger. Ovarian germ cells were isolated using a Falcon^®^ 100 μm cell strainer (Corning), connective tissue and debris were allowed to settle, obtaining a uniform single-cell suspension from ovarian tissue. The single-cell suspensions were washed twice in PBS and immediately processed for following analysis.

### 2.8. Detection of Telomeres by PNA Kit/FITC for Flow Cytometry

Detection of telomeres was performed in bone marrow cells and in ovarian germ cells of CTR, ET-FPP^®^ and LT-FPP^®^ mice obtained immediately after the sacrifice. To this purpose a Telomere PNA Kit/FITC for Flow Cytometry (Dako-Agilent, Santa Clara, CA, USA) was used. The kit allows detection of telomeres in nucleated haematopoietic cells using a fluorescence in situ hybridization and a fluorescein-conjugated peptide nucleic acid (PNA) probe. Results were evaluated by flow cytometry using a light source with excitation at 488 nm.

### 2.9. Statistical Analysis

Results in the text are reported as means ± standard error (SE), and calculation were done using the GraphPad Prism software (San Diego, CA, USA). Unpaired t-test (Student’s t-test) was applied to analyze the results. Statistical significance was set at *p* < 0.05.

## 3. Results

### 3.1. Evaluation of FPP^®^-Supplemented Water Effectiveness 

The experimental design has been set up in order to evaluate *in vivo* the effectiveness of FPP^®^ supplementation on redox balance and molecular signature of aging. Although the greater efficacy of FPP^®^ was proven when taken sublingually [28], we found that this administration was very stressful for mice [30], so we decided to dissolve FPP^®^ in the daily water.

In order to verify the real *in vivo* effectiveness of the non-orthodox FPP^®^ administration following dissolution in water, we have first evaluated the papaya antioxidant capacity when dissolved in water by a colorimetric test. Each day a sachet of FPP^®^ (3 g) was dissolved in 500 mL of water and administered to mice; each FPP^®^ mouse drank about 1 mL a day, with the resulting dose of 6 mg/mouse/day FPP^®^ taken every day. Therefore, same doses of papaya dissolved in water (500 mL for each cage) and taken by each mouse were analyzed for quantification of antioxidant power. We used a test that allowed us to estimate the total content of both hydrophilic (for example Ascorbic Acid) and hydrophobic antioxidants. As shown in Table 1, FPP^®^ in 500 mL of water had a Total Antioxidant Power of 6.7 ± 0.6 M, and in 1 mL taken by each FPP-treated mouse 13.48 ± 0.9 mM. Ascorbic acid is one of the hydrophilic antioxidants that can be quantified and, as for the Total Antioxidant Power, we measured ascorbic acid concentrations of a FPP^®^ sachet dissolved in 500 mL and of FPP^®^ dose taken by the mouse daily by fluorimetric assay. The papaya sachet in 500 mL of water had an ascorbic acid content equal to 192.2 ± 3.5 ng, while the mouse daily dose was 0.4 ± 0.03 ng (Table 1).

### 3.2. Early Treatment with FPP^®^: from 6 to 51 Weeks of Age

#### 3.2.1. Oral Administration of FFP^®^ Increases Plasma Levels of Antioxidants

**Figure 2:**
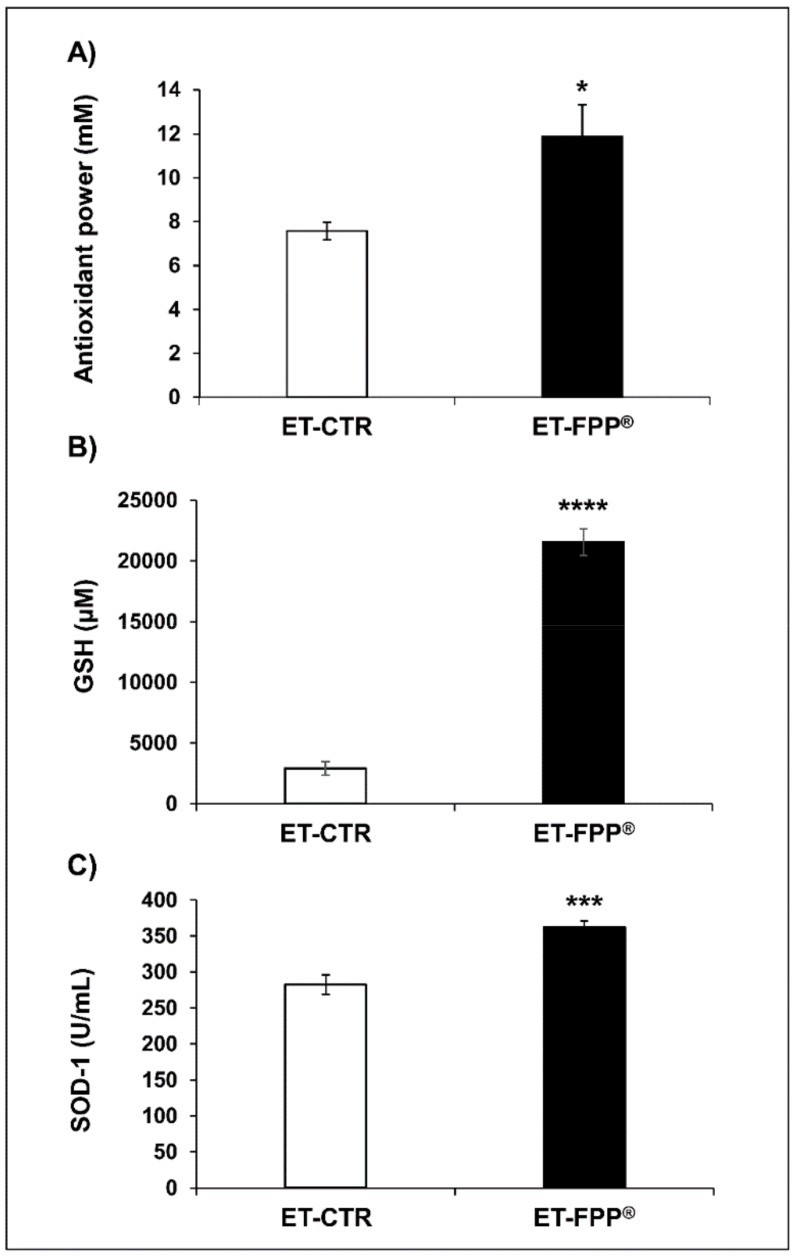
Antioxidant effect of FPP^®^ in C57BL/6J female mice by measuring the plasma antioxidant levels (antioxidant power, GSH and SOD-1). Plasma samples collected from both untreated (ET-CTR group) and treated (ET-FPP® group) mice were analyzed. (**A**) Analysis of the quantification and detection of the total antioxidant power (mM). (**B**) Analysis of the quantification and detection of GSH activity (µM). (**C**) Analysis of the quantification and detection of SOD-1 activity (U/mL). Data are normalized on total plasma and expressed as means ± SE. * *p* < 0.05, *** *p* < 0.001, **** *p* < 0.0001.

To the purpose of evaluating comparable effect in our experimental model, we first measured the Total Antioxidant Power in plasma samples of FPP^®^ in both treated and untreated mice; this test allows to detect both hydrophilic and hydrophobic antioxidant in blood samples. The results showed that mice daily treated with FPP^®^ (Figure 2A) had an increased antioxidant power (ET- FPP^®^ 11.9 ± 1.4 mM, *p* < 0.05) as compared to the control group (ET-CTR 7.6 ± 0.4 mM).

Thus, we measured the enzymatic activities of superoxide dismutase-1 (SOD-1) and plasmatic levels of reduced glutathione (GSH). SOD-1 is an enzymatic antioxidant responsible for the dissociation of superoxide anion into hydrogen peroxide and dioxygen; glutathione is a non-enzymatic antioxidant as represented by the glutathione reduced form (GSH), that plays the important role of protector against oxidative stress neutralizing reactive oxygen species. 

The results showed that ET-FPP^®^ -treated mice presented a significant increase of GSH plasmatic levels (*p* < 0.0001) of about 7.5-fold higher as compared to control plasma samples (ET-FPP^®^ 21558 ± 1100 µM, ET-CTR 2896 ± 574 µM) (Figure 2B). Comparable results were obtained with SOD-1 analysis, where SOD-1 plasmatic levels in FPP^®^ treated mice were significantly higher (ET-FPP^®^ 361 ± 9 U/mL, *p* < 0.001) as compared to control mice (ET-CTR 282 ± 13 U/mL) (Figure 2C). These results supported a potentially powerful in vivo antioxidant action exerted by FPP^®^ administered to mice from 6 weeks of age (early treatment), even when administered as dissolved in the water.

#### 3.2.2. Oral Administration of FFP^®^ Reduces Plasma Levels of ROS

**Figure 3:**
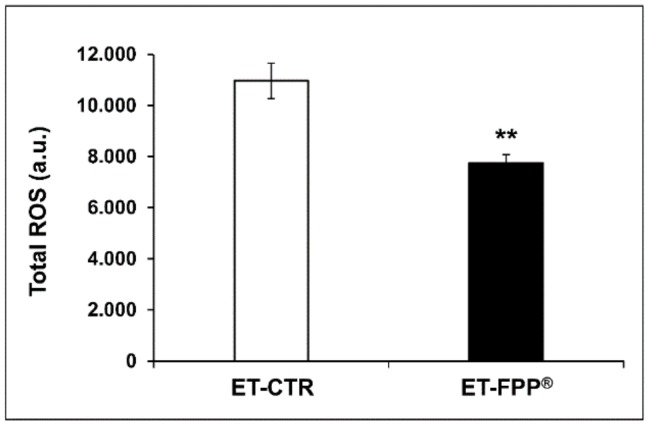
Effect of FPP^®^ on total ROS blood levels in C57BL/6J female mice. Analysis of the total ROS levels (arbitrary units, a.u.) on the plasma samples collected from both ET-CTR and ET-FPP^®^. Data are normalized on total plasma and expressed as means ± SE. ** *p* < 0.005.

Although Reactive Oxygen Species (ROS) and ROS-induced oxidative damage are not considered as the sole cause of aging, it is believed that ROS play a key role in the molecular mechanisms regulating longevity. For this reason, we evaluated and determined the effect of FPP^®^ supplementation on plasmatic levels of ROS in our experimental model. The results in Figure 3 showed a significant decrease (*p* < 0.005) of plasmatic ROS levels in ET-FPP^®^ mice (7737 ± 331 a.u.) as compared to ET-CTR group (10962 ± 692 a.u.).

#### 3.2.3. Oral Administration of FFP^®^ Increases Plasmatic Telomerase Activity

Telomerase (TE) is an enzyme that adds repetitive sequences of DNA to the chromosomal ends (telomeres); at each DNA replication, the telomeres undergo shortening and the task of telomerase is to maintain their integrity. In fact, in the absence of telomerase, the telomeres progressively shorten until they reach a threshold value where cell division stops, thus inducing cell senescence. Telomerase activity and telomeres length are currently considered the molecular signature of aging. To this purpose, we first determined and quantified the telomerase activity in the plasmas of FPP^®^-treated mice (ET-FPP^®^) as compared to the control group (ET-CTR).

**Figure 4:**
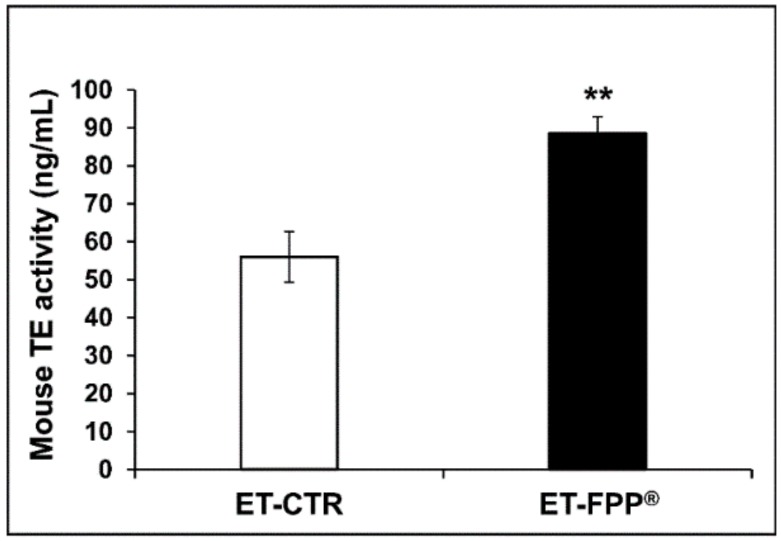
Effect of FPP^®^ on telomerase (TE) activity in plasma samples from C57BL/6J female mice. Quantitative determination of mouse telomerase (TE) activity (ng/mL) was performed on plasma samples obtained from both ET-CTR and ET-FPP^®^ groups immediately before the sacrifice. Data are normalized on total plasma and expressed as means ± SE. ** *p* < 0.005.

As shown in Figure 4, we observed an increase in telomerase concentration of mice daily treated with FPP^®^ as compared to mice drinking tap water. More in details, ET-FPP^®^ mice had a concentration of TE 1.6-fold higher (*p* < 0.005) as compared to ET-CTR (ET-FPP^®^ mice: 88.5 ± 4.5 ng/mL, ET-CTR mice: 55.9 ± 6.6 ng/mL).

#### 3.2.4. Oral Administration of FFP^®^ Increases Telomeres Length in Bone Marrow Cells and Ovarian Germ Cells

In order to evaluate the effect of FPP^®^ treatment on telomeres length, we analyzed single cell suspensions obtained from both bone marrow and ovaries of either FPP^®^ treated or untreated mice. To this purpose bone marrow and ovaries were obtained from each mouse and the single cell suspensions were isolated as described in the Materials and Methods section; subsequently bone marrow and ovarian germ cells were counted by trypan blue exclusion under optical microscope. In ET-FPP^®^ mice bone marrow and ovarian germ cells were respectively almost 4-fold and 2-fold more than the ET-CTR cells (data not shown).

**Figure 5:**
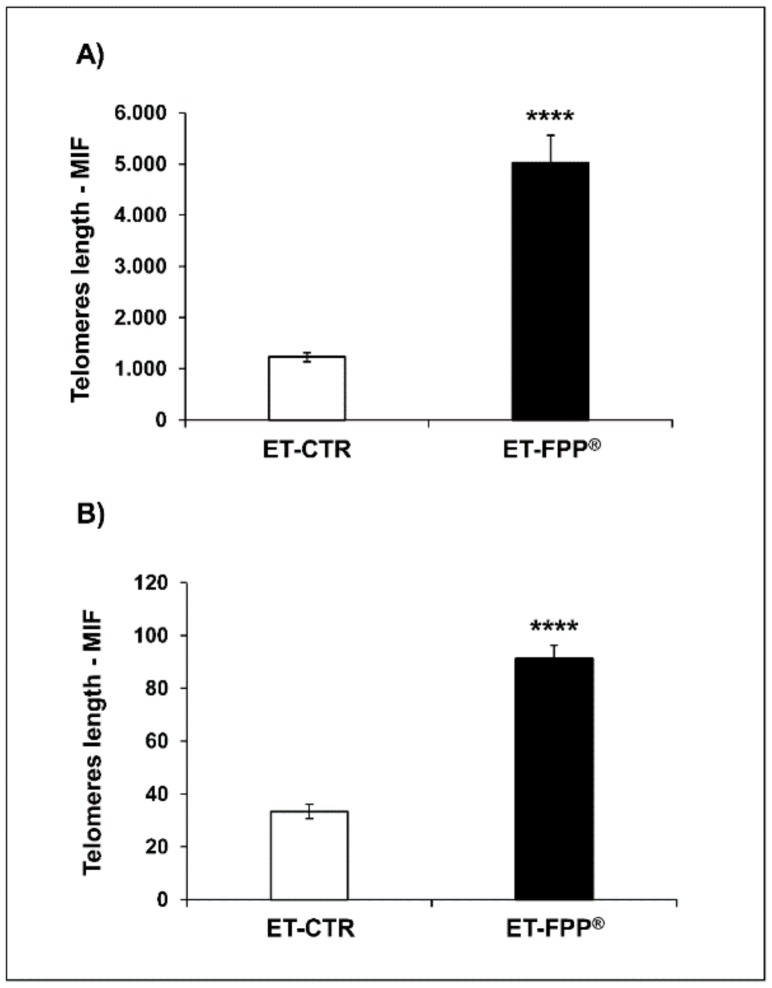
Effect of FPP^®^ on telomeres length in bone marrow cells and in ovarian germ cells from C57BL/6J female mice. The analysis of telomeres length was performed on nucleated haematopoietic cells from (**A**) bone marrow and (**B**) on ovarian germ cells. Cells were retrieved from both ET-CTR and ET-FPP^®^ groups immediately after the sacrifice. Data are expressed as mean ± SE of M.I.F. (Mean Intensity Fluorescence) normalized on total cells. **** *p* < 0.0001.

Comparable numbers of cells obtained from both organs were analyzed by hybridization of a fluorescein-conjugated probe (PNA) recognizing the sequence of six nucleotides (TTAGGG) repeated in the telomeres. The results, expressed as mean intensity of fluorescence (M.I.F.), are summarized in Figure 5. TTAGGG sequence in telomeres correlated with the value of the M.I.F.

The results showed that ET-FPP^®^ mice had an impressive increase of telomeres length than ET-CTR in both organs. In details the telomeres lenght in bone marrow cells was 4-fold higher than in control group (ET-FPP^®^: 5020 ± 542 M.I.F, ET-CTR: 1228 ± 88 M.I.F., *p* < 0.0001) (Figure 5A), while in ovarian germ cells the telomeres length was 2.7-fold higher as compared to controls (ET-FPP^®^: 91 ± 5 M.I.F., ET-CTR 33 ± 3 M.I.F., *p* < 0.0001) (Figure 5B). 

### 3.3. Late Treatment with FPP^®^: from 51 to 96 Weeks of Age

This set of experiments was aimed at evaluating the FPP^®^ anti-aging effect in a group of mice that started the treatment later in their life (10 months) (LT-FPP^®^). As for ET-FPP^®^ mice, we measured antioxidants and ROS levels and telomerase in the blood and the telomeres length in single cell suspensions obtained from the bone marrow and the ovaries of the mice.

#### 3.3.1. Oral Administration of FFP^®^ Increases Plasma Levels of Antioxidants

**Figure 6:**
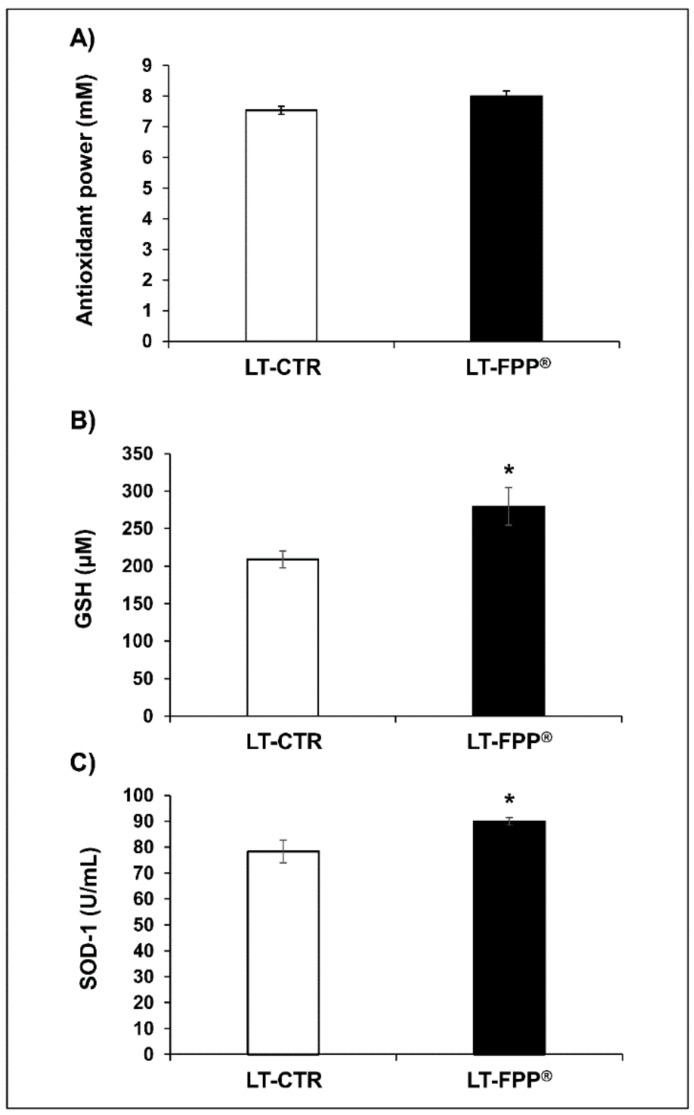
Antioxidant effect of FPP^®^ in C57BL/6J female mice by measuring the plasma antioxidant levels (antioxidant power, GSH and SOD-1). Plasma samples collected from both untreated (LT-CTR group) and treated (LT-FPP^®^ group) mice were analyzed. (**A**) Analysis of the quantification and detection of the total antioxidant power (mM). (**B**) Analysis of the quantification and detection of GSH activity (µM). (**C**) Analysis of the quantification and detection of SOD-1 activity (U/mL). Data are normalized on total plasma and expressed as means ± SE. * *p* < 0.05.

The results showed that Total Antioxidant Power levels in LT-FPP^®^ (8 ± 0.17 mM) were comparable (*p* > 0.05, not significant) to LT-CTR (7.5 ± 0.13 mM) (Figure 6A); similarly to previous results in ET-FPP^®^ mice, GSH plasmatic levels resulted to be 279.5 ± 24.9 µM in LT-FPP^®^ mice (*p* < 0.05) and 208.9 ± 11.2 µM in LT-CTR (Figure 6B), and SOD-1 levels 89.9 ± 1.5 U/mL in LT-FPP^®^ mice (*p* < 0.05) and 78.3 ± 4.3 U/mL in LT-CTR group (Figure 6C). It is clear from the figures that while the significant increase in the SOD-1 and GSH plasma levels in the FPP^®^ -treated mice, the absolute values are lower than in the group of mice that started the FPP^®^ treatment earlier in their life (Figure 2).

#### 3.3.2. Oral Administration of FFP^®^ Reduces Plasma Levels of ROS

**Figure 7:**
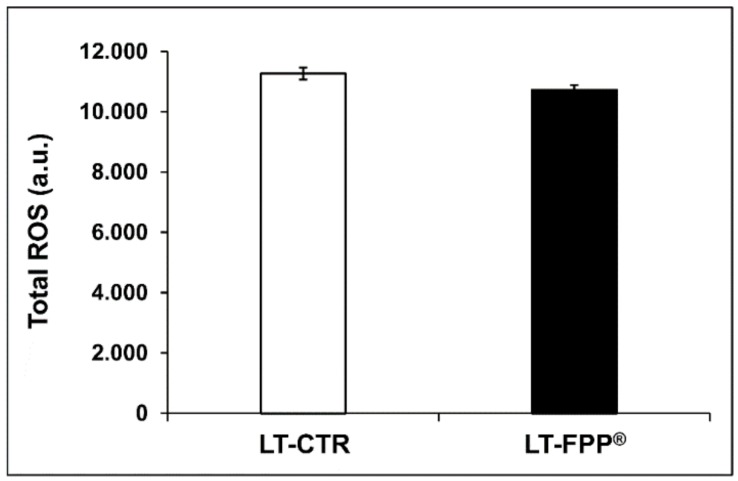
Effect of FPP^®^ on total ROS blood levels in C57BL/6J female mice. Analysis of the total ROS levels (arbitrary units, a. u.) on the plasma samples collected from both LT-CTR and LT-FPP^®^. Data are normalized on total plasma and expressed as means ± SE. *p* = NS (>0.05).

This set of results did not show significant difference in the plasmatic ROS levels between the FPP^®^-treated and untreated mice (*p* > 0.05, not significant). In fact, LT-FPP^®^ mice had ROS levels of 10727 ± 157 a.u. and LT-CTR mice 11266 ± 198 a.u. (Figure 7). 

#### 3.3.3. Oral Administration of FFP^®^ Increases Plasmatic Telomerase Activity 

**Figure 8:**
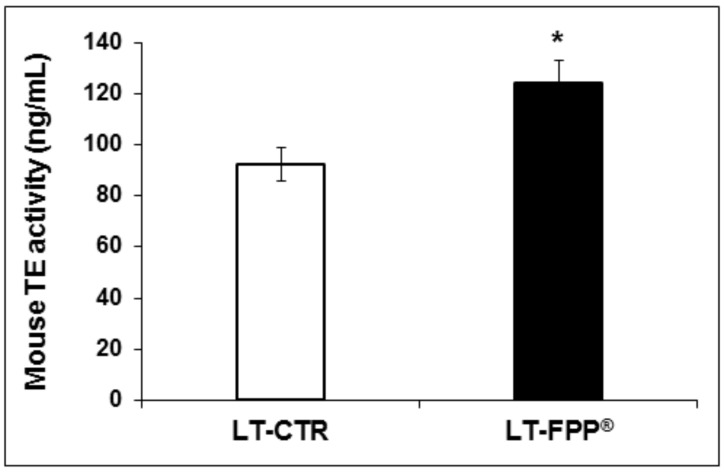
Effect of FPP^®^ on telomerase (TE) activity in plasma samples from C57BL/6J female mice. Quantitative determination of mouse telomerase (TE) activity (ng/mL) was performed on plasma samples obtained from both LT-CTR and LT-FPP^®^ groups immediately before the sacrifice. Data are normalized on total plasma and expressed as means ± SE. * *p* < 0.05.

Analysis of telomerase activity was performed also for LT-FPP^®^ mice LT-CTR controls. As for the early treatment, in this case the mice that received water supplemented with FPP^®^ showed a higher telomerase concentration (LT-FPP^®^: 124.0 ± 9.0 ng/mL, *p* < 0.05) than the mice that drank only tap water (LT-CTR: 92.5 ± 6.5 ng/mL) (Figure 8).

#### 3.3.4. Oral Administration of FFP^®^ Increases Telomeres Length in Bone Marrow Cells and Ovarian Germ Cells

**Figure 9:**
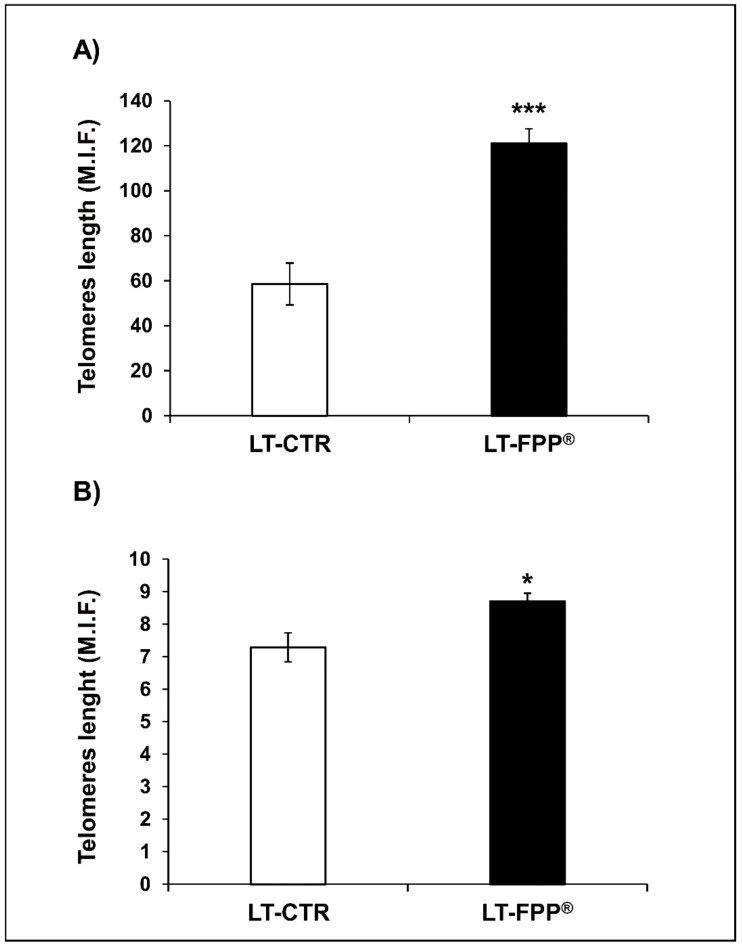
Effect of FPP^®^ on telomeres length in bone marrow cells and in ovarian germ cells from C57BL/6J female mice. The analysis of telomeres length was performed on nucleated haematopoietic cells from (**A**) bone marrow and (**B**) on ovarian germ cells. Cells were retrieved from both LT-CTR and LT-FPP^®^ groups immediately after the sacrifice. Data are expressed as mean ± SE of M.I.F. (Mean Intensity Fluorescence) normalized on total cells. * *p* < 0.05, *** *p* < 0.0005.

As previously described, bone marrow and ovarian germ cells were obtained from each mouse and hybridizated of with a fluorescein-conjugated probe (PNA) for telomeres length analysis. As for the previous group of experiments cells were counted by trypan blue exclusion under optical microscope and the results showed that the number of bone marrow and ovarian germ cells in LT-FPP^®^ mice was increased 1.8-fold and 2-fold, respectively, as compared to cells obtained from LT-CTR mice. The results on telomeres length showed that cells from the bone marrow of LT-FPP^®^ treated mice had 2-fold longer telomeres (121 ± 6 M.I.F., *p* < 0.0005) as compared to LT-CTR 59 ± 9 M.I.F. (Figure 9A). Similarly, telomeres analyzed from ovarian germ cells of LT-FPP^®^ mice were significantly longer (8.69 ± 0.25 M.I.F., *p* < 0.05) than telomeres from untreated mice (7.29 ± 0.44 M.I.F.) (Figure 9B). Consistent with the anti-oxidant reaction the M.I.F. signals in both bone marrow and ovarian germ cells were significantly decreased in LT-FPP^®^ as compared to ET-FPP^®^.

### 3.4. Comparison of FPP^®^ Effectiveness between Early Treatment and Late Treatment Supplementation

We thus wanted to compare the early to the late FPP^®^ treatment in terms of percentage of ratio between FPP^®^ -treated mice and untreated controls. As shown in Table 2, the most beneficial effects are observed with the early treatment. 

Comparing ET-FPP^®^ and LT-FPP^®^ values with their respective control, we could observe that the early treatment with FPP^®^ was impressively more effective in increasing the plasmatic levels of the antioxidant power (increase of 56%) as compared to the late treatment (1%) that was in fact comparable to CTR.

In the case of GSH the differences between early and late treatment were straightforward: in the case of ET mice GSH plasmatic level was increased of 640% compared to control, while we observed only 34% of increase in the LT group. Also SOD-1 levels were higher in the early treated mice (30%), as compared to the late treatment (15%). Moreover, FPP^®^ showed a greater effect in decreasing total ROS levels when administered early (30% of decrease) than in LT-FPP^®^ group (5% of decrease). 

Comparable results were obtained with telomerase levels (58% increase in ET-FPP^®^ mice and 34% in LT-FPP^®^) as compared to controls, and telomeres length in bone marrow and ovarian germ cells (length increase of 300% and 174% in ET-FPP^®^ and 101% and 19% in LT-FPP^®^, respectively). All in all this analysis allows to conclude that the early treatment with FPP^®^ starting from 6 weeks of life was the most effective.

## 4. Discussion

The improved living conditions, reduced rate of child mortality and advances in the medical field have led to an increased life expectancy compared to past decades. Despite this, aging and in particular age-related diseases are still major causes of mortality worldwide. Aging is characterized by a loss of fitness over time, with a series of molecular and macromolecular damages over the course of a lifetime. Faulty regulation of cellular processes could damage physiological integrity of cells and let to accumulation of damaged bioproducts. Among the various phenomena associated with aging, there is oxidative stress, characterized by the loss of balance of antioxidants/reactive oxygen species, with an accumulation of ROS at the cellular level. At the molecular level, there is a progressive shortening of the telomeres that, reached a threshold level, lead to cellular senescence and/or apoptosis. Crucial is the role of telomerase, a polymerase that can elongate telomeres by de novo addition of TTAGGG sequence repeated in telomeres.

In this study, we evaluated the effect of FPP^®^ on redox balance (antioxidant and ROS levels) together with an anti-aging effect on telomerase concentration and telomeres length in a mice model of aging treated with FPP^®^-supplemented water from 6 weeks of age (early treatment) and from 51 weeks of age (late treatment). Interestingly, we showed that FPP^®^ dissolved in water had a higher Total Antioxidant Power and it contains measurable amount of Ascorbic Acid and we were able to measure the daily dose of ascorbic acid taken by the FPP^®^-treated mice. The dual action of ascorbic acid as scavenging antioxidant and pro-antioxidant and the direct correlation between ascorbic acid dietary intake and the increased amount of antioxidants levels (i.e. glutathione) have been previously demonstrated [56,57,58,59,60].

Our data are consistent with previous works, where the correlation between dietary antioxidants from fruits and vegetables and the effects on the increase of antioxidant levels in the treated subjects has been extensively studied [9,10]. Our results showed that the daily intake of FPP^®^ significantly increased the levels of antioxidants in the blood (Total Antioxidant Power, GSH and SOD-1) and decreased the levels of total ROS, together with a clear anti-aging effect as shown by the length of telomeres and telomerase quantification in FPP^®^ treated mice. These results are consistent with previous studies where the crucial role of FPP^®^ in reducing oxidative stress and inducing the antioxidant defense response have been extensively investigated [22,23,24,25,26,27,28,29,37,38,61]. Previous reports have shown that FPP^®^ is able to modulate characteristic phenomena of elderly people, such as the pro-inflammatory profile [31] and the oxidative damage [44,45]. Moreover, FPP^®^ supplementation induced several beneficial effects in patients with neurodegenerative diseases and Electromagnetic Field Intolerance Syndrome [21,43,47,48]. Supporting these reports we have shown that plasmatic telomerase concentration and the telomeres length in bone marrow and ovarian germ cells, are significantly increased by the daily FPP^®^ administration. In our experiment, we have shown that although papaya has an effect even in beginning treatment later in life, the early treatment is far more effective. This is conceivably due to the fact that the anti-aging action of FPP^®^ in maintaining telomeres length and mitigating progressive age-related shortening, is reduced when at a later age the telomeres have reached a shorter length. These results are consistent with previous reports on the attenuation of telomere length by antioxidants during aging [62,63] and on FPP^®^ action in modulating aging mechanisms [44,45,46,47,49]. 

Similarly considering the redox balance, in ET-FPP^®^ mice we have much higher antioxidants levels and a greater reduction of ROS than in LT-FPP^®^ mice. The plasma levels of antioxidants, in particular GSH and SOD-1, usually decrease with increasing age [64,65,66,67], therefore in the case of LT-FPP^®^ mice, FPP^®^ induces an increase in GSH and SOD-1, but starting from lower basal levels, thus failing to trigger a minimally comparable anti-aging action.

## 5. Conclusions

Our results showed the beneficial effect of FPP^®^ on the redox balance and anti-aging effect in mice drinking daily FPP^®^ dissolved in water. Furthermore, our data showed that early treatment induced greater effects than late treatment. Lastly, while administered in a non-orthodox way (i.e., dissolved in water, rather than sublingually) the daily FPP^®^ intake has shown to be highly effective in increasing the body anti-oxidant reaction and in improving the molecular signatures of aging.

## Figures and Tables

**Table 1:** Total Antioxidant Power and Ascorbic Acid quantification in FPP^®^-supplemented water.

	FPP^®^ in 500 mL Water (the Amount in a Bottle for Each Cage)	FPP^®^ Drank by Mice Daily
Total Antioxidant Power	6.7 ± 0.6 M	13.48 ± 0.9 mM
Ascorbic Acid	192.2 ± 3.5 ng	0.4 ± 0.03 ng

Data are expressed as mean ± SE of three experiments.

**Table 2:** Comparison between ET- and LT-treatment.

	ET-FPP^®^	LT-FPP^®^
Total Antioxidant Power	+56%	+1%
GSH	+640%	+34%
SOD-1	+30%	+15%
Total ROS	−30%	−5%
Telomerase	+58%	+34%
Telomeres length of bone marrow cells	+300%	+101%
Telomeres length of ovarian germ cells	+174%	+19%

Results are expressed as percentage ratio between value of FPP^®^ mice and respective control.
